# Facilitating validation of prediction models: a comparison of manual and semi-automated validation using registry-based data of breast cancer patients in the Netherlands

**DOI:** 10.1186/s12874-019-0761-5

**Published:** 2019-06-08

**Authors:** Cornelia D. van Steenbeek, Marissa C. van Maaren, Sabine Siesling, Annemieke Witteveen, Xander A. A. M. Verbeek, Hendrik Koffijberg

**Affiliations:** 1 Department of Research, Netherlands Comprehensive Cancer Organisation, Godebaldkwartier 419, Utrecht, DT 3511 The Netherlands; 2 Department of Health Technology & Services Research, MIRA Institute for Biomedical Technology and Technical Medicine, University of Twente, Drienerlolaan 5, Enschede, NB 7522 The Netherlands

**Keywords:** Prediction models, External validation, Semi-automated, Breast cancer

## Abstract

**Background:**

Clinical prediction models are not routinely validated. To facilitate validation procedures, the online Evidencio platform (https://www.evidencio.com) has developed a tool partly automating this process. This study aims to determine whether semi-automated validation can reliably substitute manual validation.

**Methods:**

Four different models used in breast cancer care were selected: CancerMath, INFLUENCE, Predicted Probability of Axillary Metastasis, and PREDICT v.2.0. Data were obtained from the Netherlands Cancer Registry according to the inclusion criteria of the original development population. Calibration (intercepts and slopes) and discrimination (area under the curve (AUC)) were compared between semi-automated and manual validation.

**Results:**

Differences between intercepts and slopes of all models using semi-automated validation ranged from 0 to 0.03 from manual validation, which was not clinically relevant. AUCs were identical for both validation methods.

**Conclusions:**

This easy to use semi-automated validation option is a good substitute for manual validation and might increase the number of validations of prediction models used in clinical practice**.** In addition, the validation tool was considered to be user-friendly and to save a lot of time compared to manual validation**.** Semi-automated validation will contribute to more accurate outcome predictions and treatment recommendations in the target population.

**Electronic supplementary material:**

The online version of this article (10.1186/s12874-019-0761-5) contains supplementary material, which is available to authorized users.

## Background

Shared decision-making regarding treatment decisions is becoming a more and more important aspect of health care [1]. To support this shared decision-making, prediction models in health care are very useful [2]. Such models predict the risk of a certain outcome of disease for an individual patient, based on patient- and disease-related characteristics [3]. Potential applications of prediction models include diagnosis, prognosis, and supporting treatment decisions.

Development of prediction models is based on a derivation cohort. However, the target population in which the model can be applied is not always comparable to this derivation cohort [4]. Populations can differ in, for example, severity of disease, age distribution or presence of comorbidities. Therefore, external validation is needed to evaluate model performance and assess if it still provides reliable outcomes when applied in a different population. Ideally, the prediction model should be validated in the target population before it is used in that population.

Numerous clinical prediction models have been developed in breast cancer care, such as models predicting the risk on axillary lymph node metastases [5–11], recurrences [12–16], survival [17–20], and positive margins following breast-conserving surgery [21]. However, there are much less external validations and even fewer validations on the actual target population in which the model is used [22]. Especially the latter is of crucial importance to make sure a model predicts the outcomes of a specific patient group accurately. Unfortunately, the application of prediction models in practice is not always straightforward as exact formulae, usable nomograms, or web-based tools are not always easy to locate or may not be made available by the developers. This makes validation of prediction models a very time-consuming task, also taking into account the fact that knowledge on statistical programming is necessary.

The Netherlands Comprehensive Cancer Organisation (IKNL), host of the Netherlands Cancer Registry (NCR), and Evidencio (https://www.evidencio.com/), an online freely accessible platform for prediction models, aim to improve accessibility, reliability, validity, and transparency of prediction models in oncology routinely used in clinical practice. Hereto, Evidencio developed a tool for semi-automatic validation of prediction models. This facilitates the validation of prediction models by offering an alternative method for time-consuming manual statistical calculations, which can demand advanced statistical knowledge. By validating more prediction models in a fully transparent manner, researchers and physicians can make an evidence-based decision to choose a model that provides the most accurate predicted risk for a certain target population. This study aims to explore whether semi-automated validation with Evidencio’s validation tool can reliably substitute manual validation. This was achieved by comparing outcome results of manual with semi-automated validation, and by evaluating our experience as a user of Evidencio’s validation tool.

## Methods

### Models and datasets

To determine the reliability of Evidencio’s validation tool in different underlying models, four prediction models were selected with different underlying formulas as described below. For all of these four models, the coefficients, formulae or source codes, and all necessary variables in the target dataset were available. Two of the four selected models had an underlying logistic regression model, which is one of the most frequently used models in clinical prediction modelling. One of the four selected models had an underlying Cox regression model, and one was based on a Kaplan Meier survival estimate.

All datasets for model validation were obtained from the NCR and the inclusion population was geared to the specific development population used for the prediction models. The NCR is a nationwide population-based cancer registry including all hospitals in the Netherlands (*n* = 89). Specially trained registrars collected patient-, tumor-, and treatment-related characteristics directly from patient files based on notification by the automated pathology archive. In case a variable was missing which was needed in the selected models, this particular item was set to ‘unknown’ when possible, or otherwise the particular patient with one or more missing values was excluded. In case a variable was set to ‘unknown’, the model used a weighted average for this specific covariate. For one model (predicted probability of axillary metastasis), additional data were gathered in the hospitals on predictive variables that were not registered in the NCR. In case one of the chosen models was already manually validated using NCR data in a previous study, the same dataset as used in the previous study was used for the semi-automated validation. The datasets used for validation were fully anonymized and not traceable to individual patients, thereby guaranteeing a patient’s privacy. All statistical analyses were performed in R software, version 3.4.0 [23], unless otherwise specified.

### CancerMath (2011)

CancerMath’s breast cancer outcome calculator predicts overall survival, breast cancer-specific survival and benefit of systemic treatment (chemotherapy or endocrine therapy) for each of the first 15 years after a breast cancer diagnosis [18, 24]. The model was developed on non-metastatic breast cancer patients diagnosed between 1973 and 2004 included in the Surveillance, Epidemiology, and End Results (SEER) database. The calculator works by entering a prognostic factor profile, whereafter the software queries the database to retrieve data on patients with a matching prognostic profile and a known outcome. Subsequently, a Kaplan Meier survival curve is generated using the actual survival data of all matching patients.

The calculator includes the variables age at diagnosis, number of positive lymph nodes, tumor size, grade, estrogen receptor (ER) status, human epidermal growth factor receptor 2 (HER2) status, histological tumor type, type of endocrine therapy and type of chemotherapy. All variables were present in the NCR.

First, the CancerMath prediction model was manually validated using the Breast Cancer Treatment Outcome Calculator, since this model was not validated on a Dutch population before. From the NCR database, 8911 female patients diagnosed with breast cancer in 2003 who satisfied the inclusion criteria of the development population (a first malignant tumor with a size from 1 to 50 mm in greatest dimension and with 0 to 7 positive lymph nodes), were selected. Although CancerMath provides survival predictions over various time horizons, for our comparison we focused on one prediction: 5-year overall survival.

### INFLUENCE (2015)

INFLUENCE estimates the 5-year conditional annual risk of locoregional recurrences in early breast cancer [12]. It is a time-dependent logistic regression model, estimating the chance on a locoregional recurrence in every year following diagnosis, conditional on the number of disease-free years. The model includes the following variables: age at diagnosis, tumor size, number of positive lymph nodes, grade, ER status, progesterone receptor (PR) receptor status, multifocality, use of radiotherapy, chemotherapy, and endocrine therapy. The model was developed on patients diagnosed in 2003–2006 with primary invasive breast cancer, without distant metastasis or ingrowth in the chest wall or skin in the Netherlands. This model was externally validated on a similar patient group diagnosed in 2007–2008 (*n* = 12,308) [12]. For semi-automated validation this same dataset was used.

### Predicted Probability of Axillary Metastasis (PPAM) (2016)

This model aimed to predict the probability of axillary lymph node metastasis in patients with a positive ultrasound. The model was developed on Chinese breast cancer patients with at least one lymph node detected on ultrasound diagnosed in the Breast Center, Cancer Hospital of Shantou University Medical College. It is a logistic regression model including the following variables: lymph node diameter, cortical thickness and presence or absence of a hilum as detected by ultrasonography, histological grade, tumor size and ER status of the primary tumor [5]. In a previous validation study the NCR was enriched with the necessary data on imaging [25]. The validation population consisted of 1416 patients with a positive ultrasound diagnosed between 2011 and 2015 with T1–3N0–1 stage breast cancer in one of the six participating hospitals in the Netherlands. Patient receiving primary systemic therapy or with bilateral breast cancer were excluded. This dataset was also used for semi-automatic validation. All statistical analyses regarding the manual validation were performed in Stata version 14.1.

### PREDICT 2.0 (2017)

PREDICT predicts the 5-year and 10-year overall survival for individual patients based on several patient- and tumor-related characteristics. It also provides the expected benefits of chemotherapy, endocrine therapy and trastuzumab [17]. The model is developed on women diagnosed with non-metastatic breast cancer, treated in East Anglia from 1999 to 2003. The underlying formula of the model is a Cox proportional hazards model and it uses information on age at diagnosis, number of positive lymph nodes, presence of micrometastasis, tumor size, tumor grade, mode of detection, ER status, HER2 status, generation of chemotherapy, and KI67 status. Mode of detection and KI67 status were not available in the NCR, and were consequently set to ‘unknown’. PREDICT version 2.0 was manually validated on NCR data [26]. The validation population consisted of 8834 patients with operated, non-metastatic primary invasive breast cancer diagnosed in 2005. Patients who received primary systemic therapy or had no pathologically established tumor were excluded.

#### Comparing semi-automatic validation outcomes with manual validation outcomes

This study assessed the outcomes of Evidencio’s validation tool (version 2.5) as available between September 2016 and June 2017, in terms of discrimination and calibration [27], and compared these with the outcomes of manual validation. By using semi-automated validation, the validation procedure itself is automated. This means that in case the underlying formula of the model has been uploaded on the Evidencio platform, only the anonymized dataset (including only the variables needed for validation) has to be uploaded, and the outcomes of the validation are automatically generated by the system. As it was not the purpose of this study to judge the model performance itself (this was already done before), this study only focusses on the outcomes using both semi-automated and manual validation. The correlation between the predicted and observed mortality risk was determined by use of a calibration plot and a computation of the slope (intercept and slope). For the calibration, the model was fitted to all observations, but for graphical representation the averages of the observed outcomes were plotted against the predicted outcomes [with 95% confidence interval (CI)], grouped by deciles based on the predicted estimates. The estimates were subsequently compared with the perfect prediction line (y = x). Results were quantified in terms of the model’s intercept and slope, which were subsequently compared between semi-automated and manual validation. The model discrimination was visualized by a receiver operating characteristic curve (ROC-curve). The ROC-curve displays the sensitivity (the proportion of patients who survived and were predicted correctly) plotted against the 1-specificity (the proportion of patients who did not survive but were predicted as they would have survived). Results were quantified with the Area Under the ROC index (AUC-index) and compared between semi-automated and manual validation. The AUC can be interpreted as follows: an AUC of 1.0 means that the model has perfect discrimination, whereas an AUC of 0.5 indicates that the model predicts random change (i.e. flipping a coin) [28]. Since the primary aim of this study was to compare outcomes of semi-automated with manual validation, the validity of the considered prediction models will not be discussed, but the focus will lie on the similarities and the differences between outcomes of the two validation methods.

## Results

### The semi-automated validation tool

Evidencio’s validation tool offers users the possibility to insert existing regression models or scripts generated in the statistical software program R directly into the Evidencio platform. First, an account has to be generated using a name and e-mail address. Directly after that the validation can be started and a specific model can be selected. Thereafter, the dataset can be uploaded and by using the ‘mapping data’ functionality, users are able to quickly connect each model variable to its corresponding data item, irrespective of different underlying codes. In case of missing data, the particular patient with a missing value will be excluded, unless there is a possibility to include unknown values for certain covariates in the prediction model. Moreover, there is a possibility to compare baseline characteristics of the validation population with those of the population on which the prediction model was developed. Subsequently, the Evidencio platform will execute the validation and provide the results: both the calibration and discrimination are quantified and graphically presented in diagrams. In case the model you wish to validate is not yet available on the platform, it is possible to upload this model in case the underlying formula is available.

Evidencio provides a function to rescale observations and handle missing data (by excluding those samples). Furthermore, it is possible to compare patient characteristics of the original cohort with the validation cohort, allowing easy identification of differences between cohorts that could influence the validation outcomes. Once a validation is conducted on the Evidencio platform, it can be saved along with Medical Subject Headings (MeSH) terms, names of institutions and authors who performed the validation, references, size and context information of the validation cohort, and relevant other information like figures and tables. After validation, results can be saved for private use, shared with peers, or published for public use. The platform generates quantitative and qualitative data, the latter in the form of graphs. Both types of information can be used to report results.

### Characteristics of the validation populations

The validation population of CancerMath consisted of 8911 patients of who 7663 (86%) were alive at 5 years following diagnosis. The validation population of INFLUENCE consisted of 12,308 patients, of who 12,033 (98%) were free of locoregional recurrences within 5 years from diagnosis. The percentage of missing values of the included variables ranged between 0 and 24%. For this reason, 2656 women had to be excluded, resulting in a final validation population of 9652 patients. The validation population of PPAM consisted of 1416 patients, of who 354 (25.0%) was diagnosed with an axillary lymph node metastasis. None of the patients were excluded, as all information was actively completed in a previous study. The validation population of PREDICT consisted, after exclusion of 973 patients (10%) due to missing values for one or more variables, of 8834 patients. Of these patients, 7723 (87.4%) were still alive at 5 years following diagnosis.

### Calibration and discrimination of prediction models

Table 1 presents the main results of the manual validation compared to the semi-automatic validation in terms of calibration and discrimination. Regarding the calibration of the models, none or very small differences were observed between the two types of validation. The intercept and slope of the manually validated CancerMath model differed with 0.01 and 0.03 from the semi-automated validation, respectively. Additional file 1: Figure S1 shows the graphical representation of the calibration using both types of validation methods. Although the figure produced by Evidencio’s automated validation tool does not show the exact estimates of the predicted probabilities and that it does not provide a straight line through these estimates, it can be observed that both regression lines behave similarly.

**Table 1:** Comparison of manual and semi-automated validation using Evidencio’s validation tool

Model	Outcome	Calibration: intercept			Calibration: slope			Discrimination: AUC		
		Manual (M)	Semi-automated (S)	Difference (M-S)	Manual (M)	Semi-automated (S)	Difference (M-S)	Manual (M)	Semi-automated (S)	Difference (M-S)
CancerMath^a^ (*n* = 8900)	5-year OS	−0.02	−0.03	0.01	1.12	1.09	0.03	0.76	0.76	0
INFLUENCE^b^ (*n* = 12,308)	Conditional 5-year LRR	−0.01	−0.01	0	1.06	1.06	0	0.74	0.74	0
PPAM^c^ (*n* = 1416)	Probability of axillary metastasis	0	0	0	0.99	0.98	0.01	0.77	0.77	0
PREDICT 2.0^d^ (*n* = 8834)	5-year OS	−0.01	−0.01	0	1.03	1.02	0.01	0.80	0.80	0
	10-year OS	−0.01	−0.01	0	1.03	1.02	0.01	0.77	0.77	0

^a,b,c^ = logistic regression, ^d^ = Cox proportional hazard regression. *PPAM* Predicted Probability of Axillary Metastasis, *n* number of patients included for validation, *OS* overall survival, *LRR* locoregional recurrence, *AUC* area under the receiver operating characteristic curve

No differences were found when comparing the validation methods for the INFLUENCE model. The intercept and slope as a result of manual validation were − 0.01 and 1.06, respectively, which were equal when executing the semi-automated validation. Additional file 1: Figure S2 shows the graphical representation of these results. As Evidencio provides standard axis labels, the figures do not match completely, but when zooming in on the concerning axis labels, one can see that the regression lines using both types of validation are similar.

The intercepts of manual and semi-automated validation when validating the Predicted Probability of Axillary Metastasis (PPAM) model were both 0. The slope of this model was 0.99 after manual validation, and 0.98 after semi-automated validation. Additional file 1: Figure S3 shows similar regression lines for both the manual and the semi-automated validation procedure. The non-linear line produced by Evidencio is a reflection of the predicted estimates that are grouped by quintiles, as can be seen in the manual validation.

For PREDICT 2.0, two outcomes were compared. First, the intercepts of the 5-year overall survival prediction using manual and semi-automated validation were both − 0.01. The slope of 5-year overall survival using the manual validation was 1.03, whereas it was 1.02 when semi-automatically validated. For 10-year overall survival, manual and semi-automated validation resulted in exactly the same intercepts and slopes as for 5-year overall survival (Table 1). Additional file 1: Figure S4 shows the results for the 5-year overall survival predictions. The AUC indices for manual and semi-automated validations of all models were identical (Table 1).

## Discussion

This study shows negligible differences between semi-automated and manual validation for each of the four models subjected to validation. Based on these four validation studies, it can be stated that Evidencio, in the current stage of development, is already a reliable substitute for manual validation for several models: we tested three frequently used logistic regression models and one frequently used Cox proportional hazard regression model). Minor differences were observed between the intercept and slopes between both validation methods. These differences are presumably due to small variations upon allocation of the validation cohort into deciles. The clinical relevance of these small variations, however, is considered to be trivial, as they do not result in a different assessment of the model’s performance. The largest advantage of the semi-automated validation tool is the fast acquirement of results. The Evidencio platform executes the analyses automatically using the underlying formula of the prediction model. This means that the whole process of translating the formula or underlying script to your own dataset in a statistical program can be avoided. This saves a lot of time and requires less experience with statistical packages in which validation is generally performed. In total, the number of actions needed to validate a model making use of the Evidencio platform appears to be less than for manual validation, especially if validation scripts are not available but need to be written as part of manual validation. The exact number of actions is, however, hard to quantify. This largely depends on the statistical programming skills (for manual validation) and experience of the researcher. The possibility to insert existing regression models or scripts generated in the statistical software program R directly into the Evidencio platform makes it usable for a wide variety of prediction models. All steps that need to be taken before making a model available online (i.e. publishing the model itself) are clearly described, thereby contributing to a high level of usability.

As the semi-automated validation method is easy to understand, reduces time as compared to manual validation, and allows fast publication of the results on the platform, it may encourage other researchers and clinicians to validate prediction models that are used in clinical practice. This is of high relevance, since prediction models are predominantly based on retrospectively collected data from a specific population. Using such a model on a different population with different characteristics may not lead to the same results by default. It is therefore important to validate a prediction model in the target population in which the model will be applied. In addition, the availability of tools that ease validation procedures may facilitate evidence-based decision-making strategies through knowledge about validity of the models. It has been shown that prediction models used in clinical practice often overestimate survival in younger [29, 30] and older patients [31]. The possibility to make validation results publicly available on the Evidencio platform may facilitate repeated validations of the same model on different subpopulations, including the youngest and the elderly, thereby contributing to the need for more external validations [32]. Revealing specific subpopulations, on which a model may not perform accurately, may encourage researchers and clinicians to update the existing model to improve its accuracy for that specific population.

An important condition for using the semi-automated validation is the availability of the underlying formula of the model, so it can be published on the Evidencio platform. As not all model developers make their underlying statistics available, it is currently not possible to include all existing prediction models on the website and consequently it is not possible to validate all existing models. However, validating existing prediction models of which the underlying formula of the model is available may already give increased insight in the performance of certain models in different populations. In the era of Findable, Accessible, Interoperable, Reusable (FAIR) data [33], we expect that more and more researchers will make the underlying formula of their model available, which will consequently lead to extension of the Evidencio platform. Furthermore, it is of vital importance to maintain the privacy of all patients included in the validation population. This is achieved as follows. First, the dataset that has to be uploaded only has to include the variables needed for the validation. None of the models needed information on patient level which would be highly identifiable. In this way, no identifiable information will be available online. Second, the data will only be saved temporarily for the user during the validation itself, and can be deleted afterwards. It will not be available for others. This way of handling data safeguards the privacy of patients and will facilitate validation procedures in a safe way.

The maximum number of observations allowed by Evidencio to analyze simultaneously as part of a model validation was limited to 10,000 at time of performing these analyses. Another possible limitation of the semi-automated validation procedure is the fact that it produces figures which cannot (yet) be adapted to one’s personal style, and that it only provides results on calibration (intercept and slot of regression model) and discrimination (AUC index). Different types of measures to assess the accuracy of a prediction model are described, which reflect different elements of performance, such as the Brier score (for binary or categorical outcomes) or the Hosmer-Lemeshow test (for binary outcomes) for overall performance, or reclassification measures (e.g. Net Reclassification Index) and decision-analytic measures (e.g. decision curve analysis) [33]. However, a calibration plot with its accompanying model intercept and slope, and a ROC curve with its accompanying AUC, as provided by the semi-automated validation, comprise the key outcome elements of model validation [34, 35]. Adding the aforementioned additional outcomes, however, would be of value to allow assessing differences between observed and predicted outcomes in even more detail. Another possible limitation is the fact that Evidencio does not have a tool yet that makes it possible to impute missing data. In our study, we did not have to exclude many patients with missing values for certain variables, so we do not expect that our results would have been biased. However, in many other countries, collected data can be less complete than in the Netherlands, resulting in more biased outcomes. A feature that makes it possible to impute missing data may lead to more accurate estimates. Furthermore, it would be of added value to have uncertainty estimates around the predicted outcomes of every model. For now, only the INFLUENCE model provides these. It is encouraged to include these uncertainty measures in any future (updates of) models.

## Conclusions

This study shows that semi-automated validation using Evidencio’s validation tool can be a good substitute for manual validation. Results regarding model calibration and discrimination for both manual and semi-automated validation were almost exactly identical and any observed differences did not alter the interpretation of the model accuracy. As we evaluated different underlying model structures, the results of this study can be generalized to other models and different populations. The Evidencio platform was considered to be very user-friendly, and its semi-automated validation tool allows researchers and clinicians to save lots of time as compared to a manual validation. The availability of semi-automated validation may increase the number of validation studies of prediction models used in clinical practice, thereby contributing to more accurate outcome predictions and treatment recommendations.

## Additional files

Additional file 1: Graphical representation of the validation studies executed by semi-automated and manual validation. The file consists of four supplementary figures. **Figure S1.** represents the calibration and discrimination of CancerMath’s prediction tool for 5-year overall survival, executed by both validation methods. **Figure S2.** shows the calibration and discrimination of the INFLUENCE prediction tool for the 5-year risk on a locoregional recurrence, executed by both validation methods. **Figure S3.** shows the calibration and discrimination of the PPAM prediction tool for the risk on axillary lymph node metastasis for both validation methods. **Figure S4.**s shows the calibration and discrimination of PREDICT’s prediction tool for 5-year overall survival, for both types of validation. (PDF 744 kb)

## Data Availability

The datasets generated and/or analysed during the current study are not publicly available due to strict privacy regulations of the Netherlands Cancer Registry. For more information please contact the corresponding author.

## Abbreviations

AUC: Area Under the Receiver operating characteristic curve
CI: Confidence interval
ER: Estrogen receptor
HER2: Human epidermal growth factor receptor 2
IKNL: Netherlands Comprehensive Cancer Organisation
MeSH: Medical Subject Headings
NCR: Netherlands Cancer Registry
PPAM: Predicted probability of axillary metastasis
PR: Progesterone receptor
ROC: Receiver Operating Characteristic
