# Supplementary material for: Facilitating validation of prediction models: a comparison of manual and semi-automated validation using registry-based data of breast cancer patients in the Netherlands
Source: BMC Med Res Methodol. 2019 Jun 8;19:117. doi: 10.1186/s12874-019-0761-5 (PMC6556016; doi:10.1186/s12874-019-0761-5)
Supplement: Supplementary file 1 — Graphical representation of the validation studies executed by semi-automated and manual validation. The file consists of four supplementary figures. Figure S1. represents the calibration and discrimination of CancerMath’s prediction tool for 5-year overall survival, executed by both validation methods. Figure S2. shows the calibration and discrimination of the INFLUENCE prediction tool for the 5-year risk on a locoregional recurrence, executed by both validation methods. Figure S3. shows the calibration and discrimination of the PPAM prediction tool for the risk on axillary lymph node metastasis for both validation methods. Figure S4.s shows the calibration and discrimination of PREDICT’s prediction tool for 5-year overall survival, for both types of validation. (PDF 744 kb) [file 12874_2019_761_MOESM1_ESM.pdf]

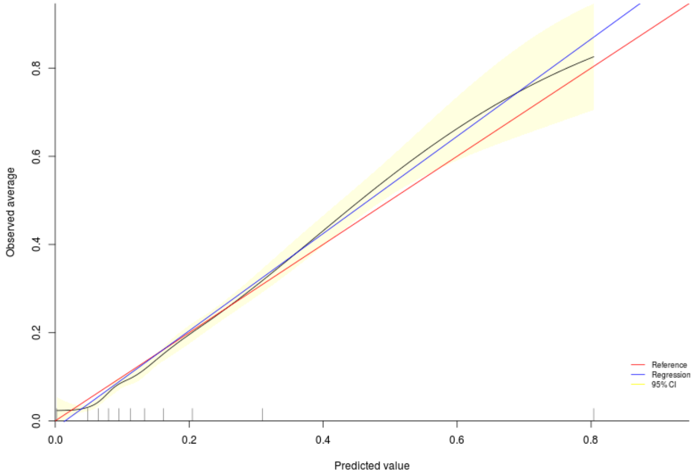

Intercept = -0.03, slope = 1.09

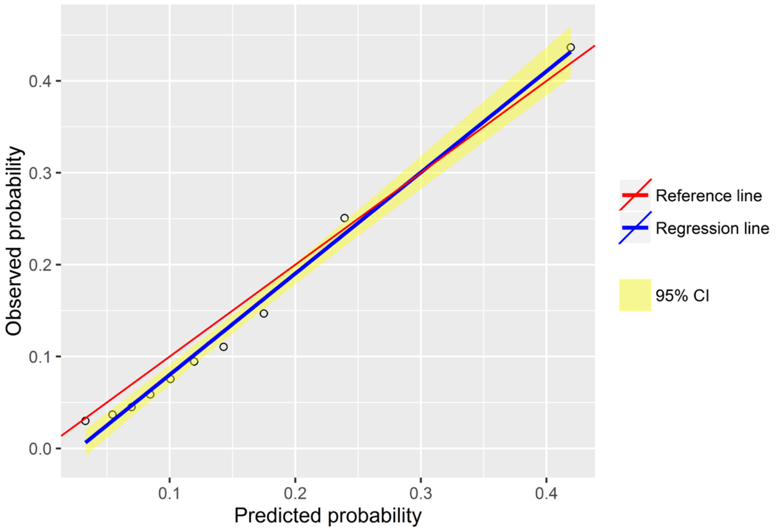

Intercept =-0.02, slope = 1.12

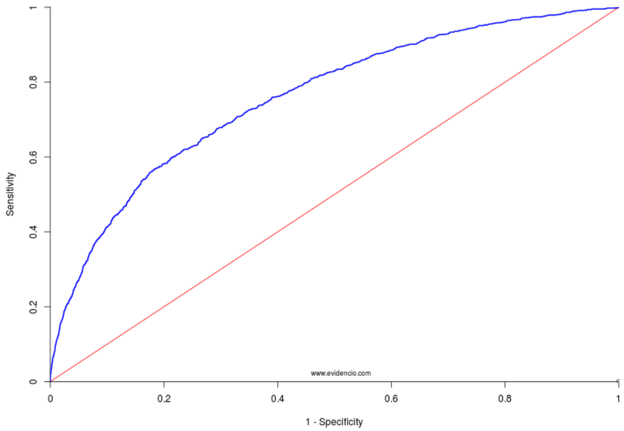

Area under the curve = 0.76

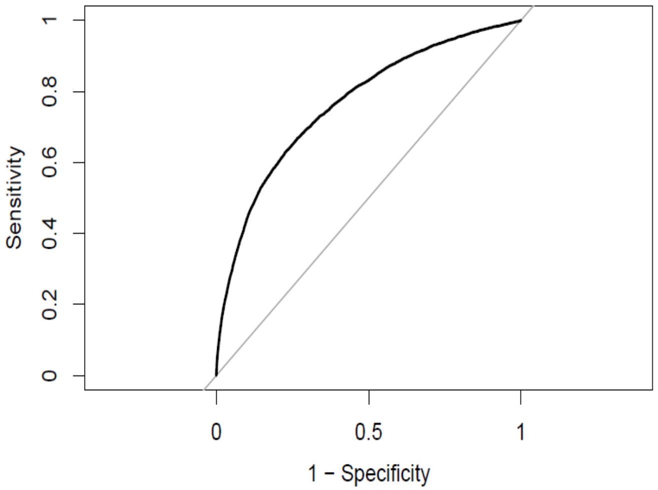

Area under the curve = 0.76

Supplementary Figure 1. Calibration and discrimination of CancerMath's prediction tool for 5-year overall survival. The left panels show the results of the semi-automated validation using Evidencio's validation tool, the right panels show the results of the manual validation in R. Please note the different distribution of axes for the calibration figures.

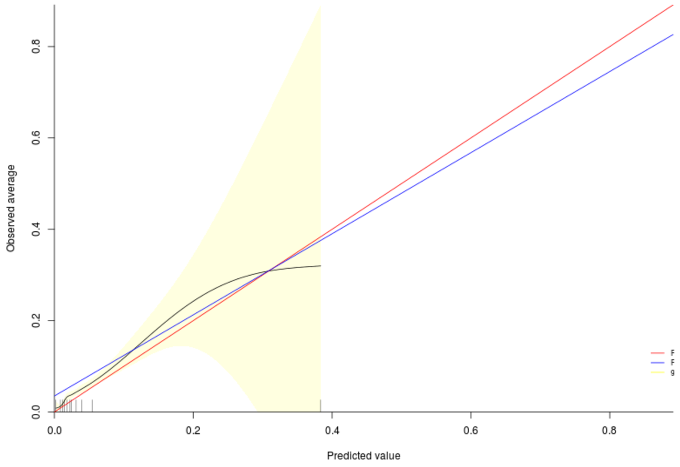

Intercept = -0.01, slope = 1.06

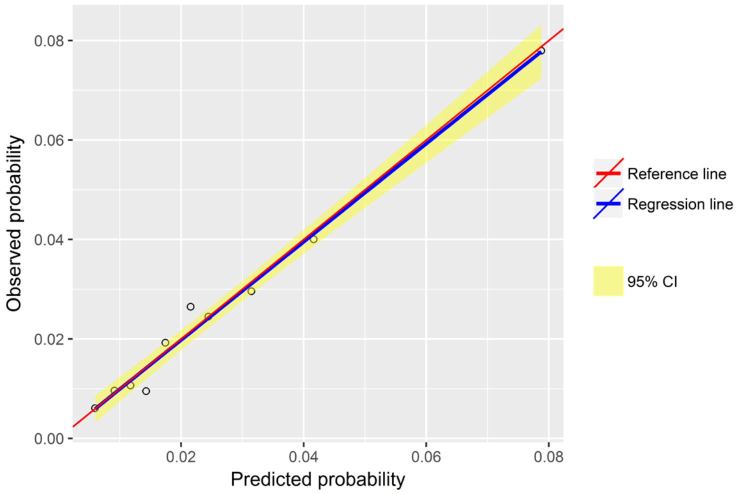

Intercept =-0.01, slope = 1.06

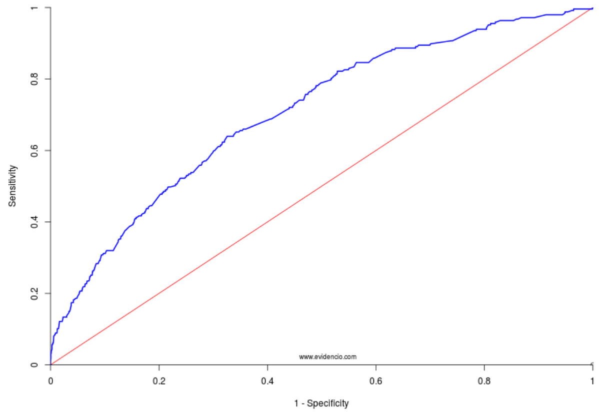

Area under the curve = 0.74

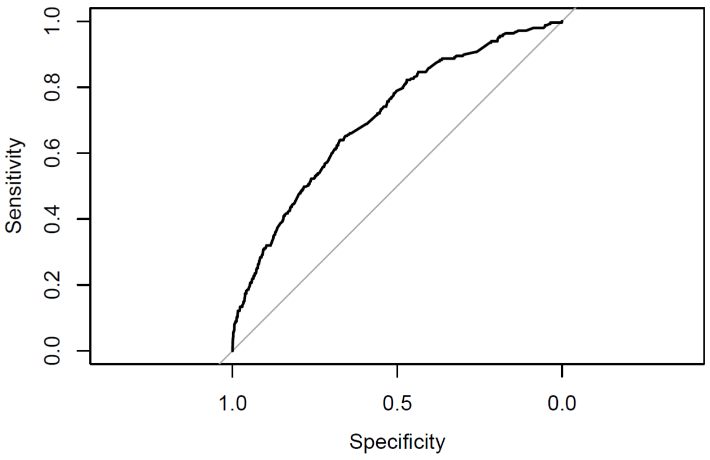

Area under the curve = 0.74

Supplementary Figure 2. Calibration and discrimination of the INFLUENCE prediction tool for the 5-year risk on locoregional recurrence. The left panels show the results of the semi-automated validation using Evidencio's validation tool, the right panels show the results of the manual validation in R. Please note the different distribution of axes for the calibration figures.

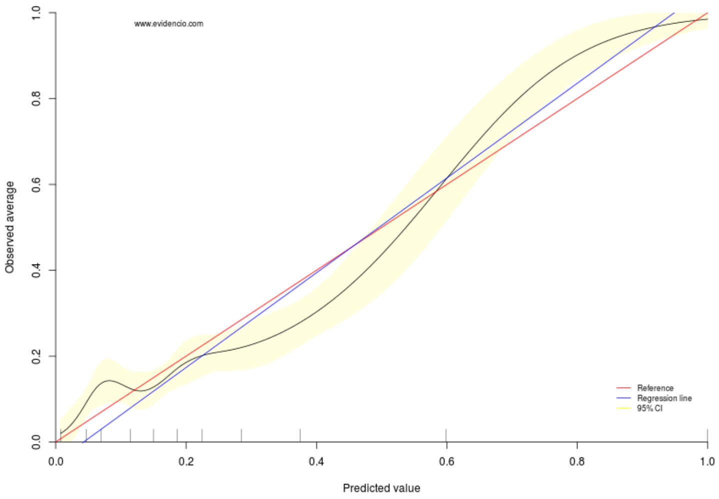

Intercept = 0, slope = 0.98

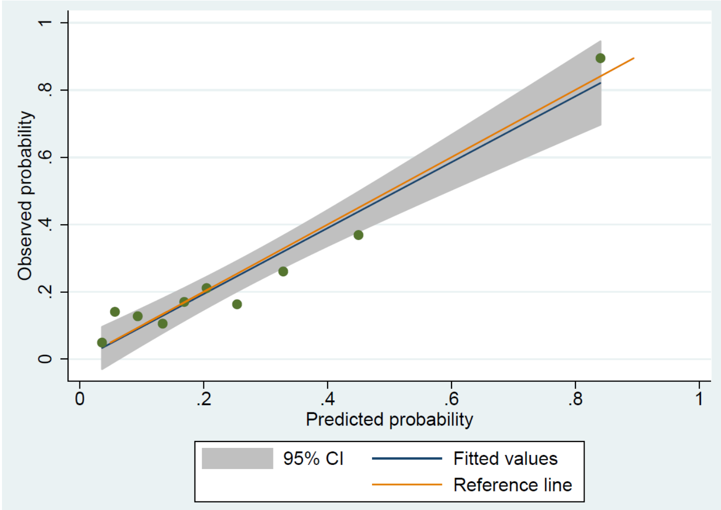

Intercept = 0, slope = 0.99

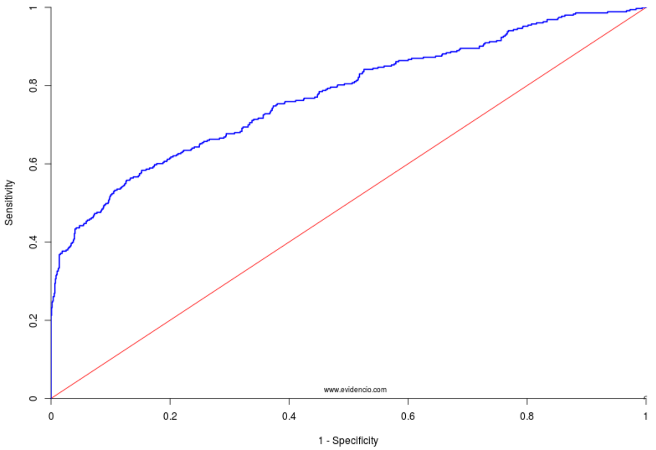

Area under the curve = 0.77

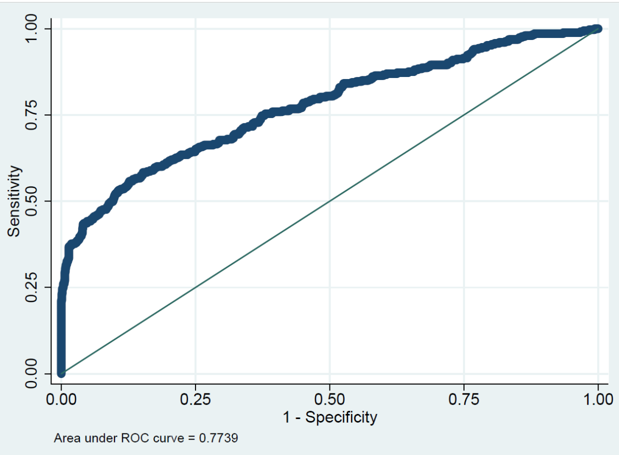

Area under the curve = 0.77

Supplementary Figure 3. Calibration and discrimination of the PPAM prediction tool for the risk on axillary lymph node metastasis. The left panels show the results of the semi-automated validation using Evidencio's validation tool, the right panels show the results of the manual validation in Stata.

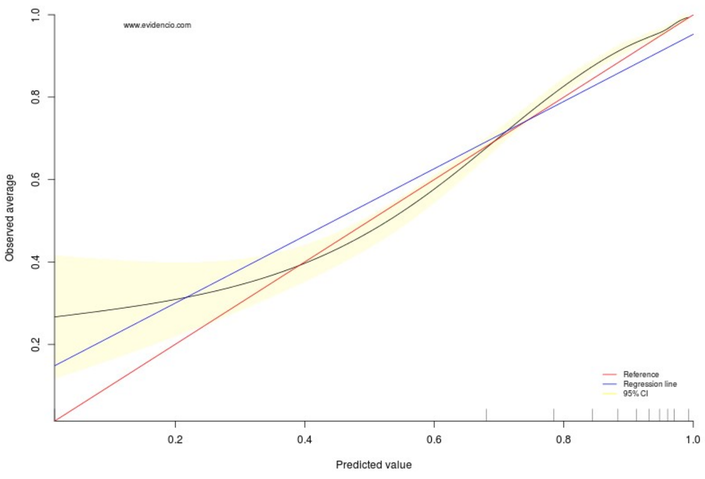

Intercept = -0.01, slope = 1.03

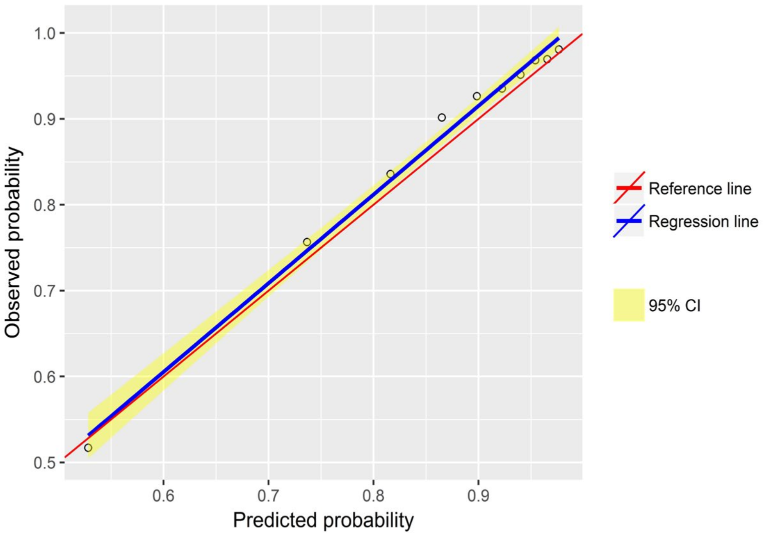

Intercept: = -0.01, slope = 1.02

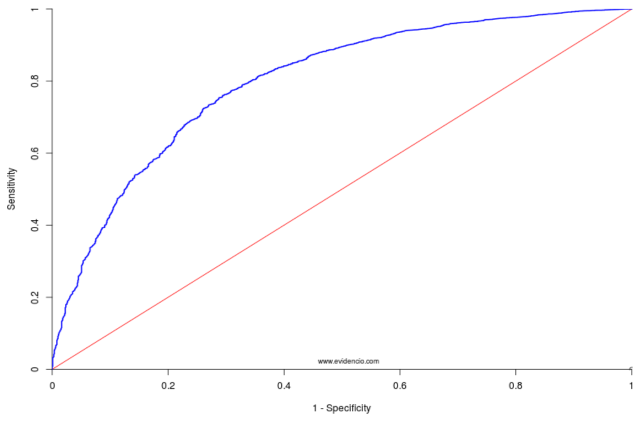

Area under the curve: 0.80

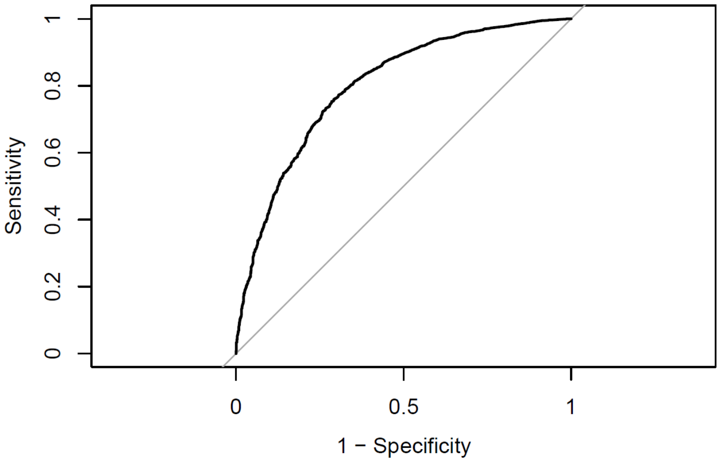

Area under the curve 0.80

Supplementary Figure 4. Calibration and discrimination of PREDICT's prediction tool for 5-year overall survival. The left panels show the results of the semi-automated validation using Evidencio's validation tool, the right panels show the results of the manual validation in R. Please note the different distribution of axes for the calibration figures.
